# Performance Investigation of AlGaInP Light-Emitting Diodes

**DOI:** 10.3390/nano16080480

**Published:** 2026-04-17

**Authors:** Weiwei Sun, Shaobo Ge, Junyan Li, Lujun Shen, Xinyu Zhao, Ronghua Shi, Jin Zhang, Yingxue Xi

**Affiliations:** Shaanxi Province Key Laboratory of Thin Films Technology and Optical Test, School of Optoelectronic Engineering, Institute for Interdisciplinary and Innovation Research, Xi’an Technological University, Xi’an 710021, China; m13821793280_1@163.com (W.S.); lijunyan@st.xatu.edu.cn (J.L.); shenlujun@st.xatu.edu.cn (L.S.); zhaoxxyuu@163.com (X.Z.); shironghua@st.xatu.edu.cn (R.S.); j.zhang@xatu.edu.cn (J.Z.); xiyingxue@xatu.edu.cn (Y.X.)

**Keywords:** multiple quantum wells (MQWs), well-layer strain, Micro-LED arrays, external quantum efficiency (EQE)

## Abstract

Previous studies have shown that the external quantum efficiency (EQE) of conventional red Micro-Light emitting diodes(Micro-LEDs) decreases markedly with reducing chip size. This degradation is generally attributed to enhanced non-radiative recombination at sidewall defects, which leads to increased carrier loss in size-scaled LEDs. In this work, AlGaInP quaternary semiconductor epitaxial wafers incorporating multiple quantum wells (MQWs) with different well-layer strain states were grown by metal–organic chemical vapor deposition (MOCVD). Through wafer bonding, photolithography, etching, and metal evaporation, these epitaxial structures were fabricated into Micro-LED arrays with single-pixel pitches of 10, 20, 50, and 100 μm. The experimental results reveal that, with increasing indium (In) composition in the GaInP well layers—corresponding to a gradual increase in lattice mismatch (Δa/a) from 0% to 1%—smaller-sized Micro-LED arrays exhibit superior EQE performance. For devices with a pixel pitch of 10 μm, the EQE of Micro-LED arrays with a 1% lattice mismatch in the well layer is approximately three times higher than that of lattice-matched (0%) counterparts. In contrast, for devices with a pixel pitch of 100 μm, the EQE of lattice-matched (0%) Micro-LED arrays is about 1.3 times higher than that of devices with a 1% lattice mismatch. These results indicate that, to achieve maximum EQE in Micro-LEDs, the strain state of the MQW-layer material must be carefully considered as a priority factor. Optimal device performance requires appropriate matching between LED size and the well-layer growth strain.

## 1. Introduction

In recent years, materials used for light-emitting diodes (LEDs) have been predominantly based on III–V compound semiconductors. In mature self-emissive LED display technologies, blue and green pixels are typically fabricated using InGaN materials, whereas red pixels rely on AlGaInP materials due to the relatively low efficiency of InGaN in the red spectral region [1]. AlGaInP quaternary semiconductors are currently the most widely used materials for red LEDs, with well-established fabrication processes. Benefiting from continuous advances and technological breakthroughs in metal–organic chemical vapor deposition (MOCVD), AlGaInP-based LEDs have become the most efficient light-emitting devices in the red–yellow spectral range at present [2].

However, conventional etching processes introduce a large density of surface states and non-radiative recombination centers, leading to a significant reduction in the luminous efficiency of Micro-LEDs. Early studies demonstrated that AlGaInP-based red LEDs possess a longer minority carrier diffusion length than GaN-based devices, resulting in a higher non-radiative recombination rate at etched sidewalls and a pronounced degradation of device efficiency as the chip size decreases [3]. Consequently, current optimization strategies primarily focus on post-etching sidewall defect passivation, including chemical treatments, thermal annealing, and passivation layer deposition. Nevertheless, these approaches only provide partial recovery of device performance [4].

In recent years, AlGaInP/GaInP heterostructures have been extensively investigated as a barrier and well layers in MQWs, respectively, owing to their ability to achieve lattice-matched growth on GaAs substrates with high crystalline quality. With the development of band-engineering techniques, strain modulation in heterostructures has been shown to significantly alter the band structure of materials and, consequently, their optoelectronic properties. For example, strain-compensated multiple quantum well (SC-MQW) structures have been widely applied in various LED devices [5,6].

In this study, we focus instead on optimizing the growth structure of multiple quantum wells (MQWs) to enhance the internal quantum efficiency (IQE), thereby improving the external quantum efficiency (EQE) of Micro-LEDs. Here, epitaxial wafers with different MQW-layer strain conditions were grown by MOCVD. At room temperature, photoluminescence (PL) measurements were performed to characterize optical parameters of emission spectra, while high-resolution X-ray diffraction (HRXRD) was used to analyze the diffraction peaks of each experimental wafer. After fabrication into Micro-LED arrays, devices with different well-layer strain states were characterized by electroluminescence (EL) measurements to obtain EQE–current characteristics and current–voltage (I–V) curves. To ensure the accuracy of the test data, a one-minute waiting period was implemented after each measurement condition, allowing the sample to return to thermal equilibrium.

## 2. Experimental Methods

AlGaInP red-emitting epitaxial wafers with different well-layer strain states were grown on (100)-oriented GaAs substrates miscut by 15° toward <111> using a metal–organic chemical vapor deposition (MOCVD) system (Aixtron G4-2800 (Aixtron SE., Herzogenrath, Germany)). Trimethylaluminum (TMAl), trimethylgallium (TMGa), and trimethylindium (TMIn) were employed as group-III precursors, while arsine (AsH_3_) and phosphine (PH_3_) served as group-V sources. Disilane (Si_2_H_6_) and bis(cyclopentadienyl)magnesium (Cp_2_Mg) were used as n-type and p-type dopant sources, respectively [7]. High-purity H_2_ was used as the carrier gas to transport the precursors into the reactor for epitaxial growth.

**Figure 1:**
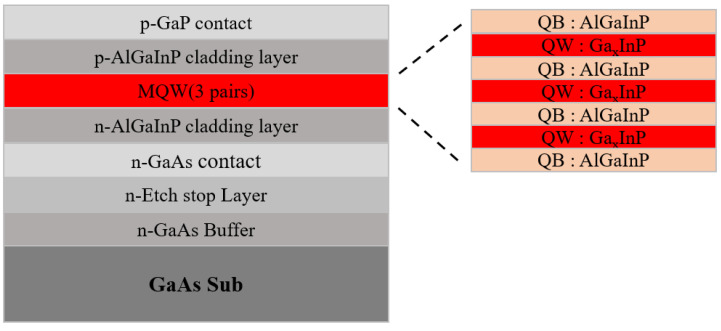
Schematic of AlGaInP Epi structure.

A schematic illustration of the epitaxial structure is shown in Figure 1, and the detailed layer structure is described as follows. A ~300 nm thick Si-doped GaAs buffer layer was first grown on the substrate, followed by a ~300 nm thick Si-doped Ga_0.5_In_0.5_P etch-stop layer. Subsequently, a ~50 nm thick heavily Si-doped n^+^-GaAs contact layer was deposited. An n-type (Al_0.7_Ga_0.3_)_0.5_In_0.5_P layer with a thickness of ~300 nm was then grown with a Si doping concentration of approximately 1 × 10^18^ cm^−3^. The active region consisted of three periods of (Al_0.7_Ga_0.3_)_0.5_In_0.5_P/Ga_0.5_In_0.5_P multiple quantum wells (MQWs), and the adoption of a three-pair MQW design is motivated by the increased sidewall area ratio as the device size decreases; consequently, a reduced number of QW pairs can minimize the sidewall exposure area, thereby decreasing non-radiative recombination of carriers. A p-type (Al_0.7_Ga_0.3_)_0.5_In_0.5_P layer with a thickness of ~300 nm and Mg-doped at a concentration of ~1 × 10^18^ cm^−3^ was subsequently deposited, followed by a 300 nm thick Mg-doped GaP contact layer.

To investigate the influence of GaInP well-layer strain, three different GaInP well compositions were designed, as summarized in Table 1. As the In composition in the well layer increases, both the emission wavelength and the stress induced by the lattice mismatch change, where Δa/a represents the degree of the lattice mismatch. To maintain a consistent emission wavelength across the epitaxial wafers for performance comparison, it is necessary to appropriately reduce the well thickness when increasing the In composition in the well layer. In the base condition, the In composition in the GaInP well was 0.5, corresponding to an approximately lattice-matched structure with a well thickness of 6 nm. In experimental condition 1, the In composition was increased to 0.55 with a well thickness of 5 nm, resulting in a lattice mismatch (Δa/a) of 0.5%. In experimental condition 2, the In composition was further increased to 0.6 with a reduced well thickness of 4 nm, corresponding to a lattice mismatch of 1%. The barrier layers in all three experimental groups were grown from the same (Al_0.7_Ga_0.3_)_0.5_In_0.5_P composition, with a thickness of 7 nm. The MQW growth was conducted at a pressure of 50 mbar, with the reactor temperature maintained at 660 °C. Epitaxial wafers grown under these conditions were characterized by photoluminescence (PL (ZTO, Beijing, China)) and X-ray diffraction (XRD (Bruker, Billerica, MA, USA)) to evaluate emission wavelength and crystalline quality.

**Figure 2:**
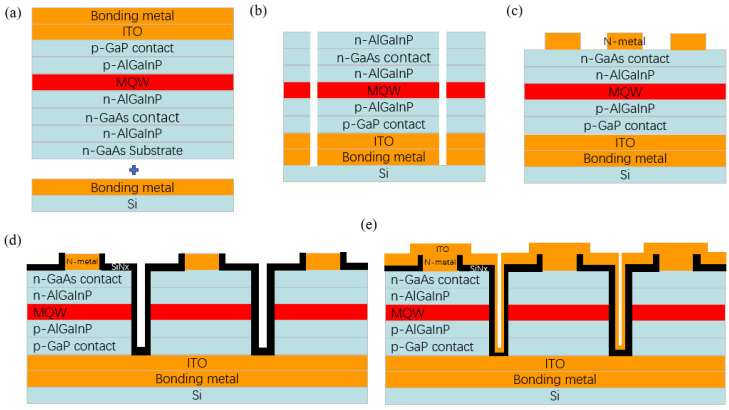
Schematic illustration of the Micro-LED array fabrication process: (**a**) wafer bonding, (**b**) GaAs substrate removal and die separation, (**c**) n-type metal deposition, (**d**) mesa formation and pixel isolation, (**e**) n-side electrode interconnection.

To further analyze the performance impact induced by different well-layer strain conditions, epitaxial wafers grown under the three experimental conditions were fabricated into Micro-LED display arrays with four different pixel pitches, namely 10, 20, 50, and 100 μm in diameter. The fabrication process is schematically illustrated in Figure 2. First, the epitaxial wafer was bonded to a Si-based backplane via metal deposition and wafer bonding, as shown in Figure 2a. The metal stack on the epitaxial wafer consists of Cr_10_Au_1500_Pt_400_Sn_600_Au_600_, and the metal stack on the backside consists of Ti_50_Pt_300_Au_100_. The GaAs substrate and buffer layer were then removed by wet chemical etching. The solution used was a mixture of ammonium hydroxide (NH_4_OH) and hydrogen peroxide (H_2_O_2_) at a volume ratio of 1:7. After photolithographic definition of isolation channels, a combination of wet and dry etching was employed to form trenches and separate individual dies, as shown in Figure 2b. AuGeNi was deposited on the n^+^-GaAs contact layer to form the n-type electrodes (Figure 2c). Subsequently, ICP-RIE etching was used to etch through the epitaxial layers, enabling pixel isolation and the formation of Micro-LED arrays with pixel pitches of 10, 20, 50, and 100 μm, as shown in Figure 2d. The etched sidewalls were passivated by Si_3_N_4_ deposition, followed by the deposition of indium tin oxide (ITO) to achieve n-side interconnection of the emitting pixels, as illustrated in Figure 2e. Upon completion of the fabrication process, the Micro-LED arrays were characterized at room temperature using an electroluminescence (EL(FitTech, Shenzhen, China)) measurement system to obtain current–voltage (I–V) characteristics and EQE–current curves. In addition, reliability tests were conducted using an integrating sphere-based optoelectronic parameter analyzer under a current density of 70 A cm^−2^ at 60 °C and 90% relative humidity for 500 h.

## 3. Results and Discussion

### 3.1. Emission Wavelength and Crystalline Quality of the Epitaxial Wafers

**Figure 3:**
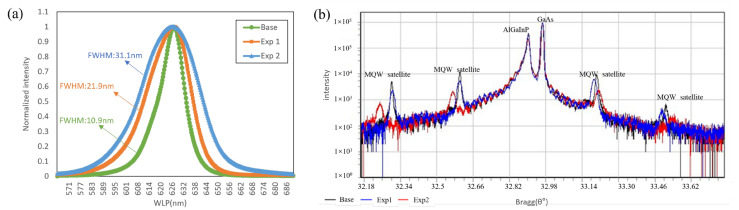
(**a**) Emission peak spectra, (**b**) XRD peaks.

Figure 3a shows the photoluminescence (PL) emission spectra of the as-grown epitaxial samples, measured using a 532 nm laser as the excitation source. The emission intensity was normalized for comparison. As the In composition in the GaInP well layers increases, the strain induced by lattice constant mismatch becomes more pronounced, resulting in a gradual broadening of the full width at half maximum (FWHM) of the emission spectra. When the In composition in the GaInP well layer reaches 0.6, corresponding to a strain level of 1%, the FWHM increases from 10.9 nm to 31.1 nm. This behavior can be attributed to the increase in In composition within the GaInP well, which leads to a reduction in the bandgap. As the In content rises, the bandgap (Eg) of the GaInP well narrows, resulting in a deeper potential well. To maintain consistent emission wavelength, the well thickness is correspondingly reduced. The appropriate reduction in quantum well thickness results in an increased separation between the quantized energy states (ground and excited states) within the well, thereby leading to a corresponding shift toward shorter wavelengths. Consequently, as the indium composition in the GaInP quantum well increases, fluctuations in indium concentration can cause broadening of the photoluminescence (PL) spectrum. However, we still need to verify the impact of increased In composition on crystal quality.

Figure 3b presents the X-ray diffraction (XRD) rocking curves of the epitaxial layers. The diffraction peak of the GaAs substrate is clearly observed, while the AlGaInP diffraction peak appears on the left side. This peak corresponds to the cladding layer. Although the main MQW peak is partially overlapped by the AlGaInP peak, four satellite peaks of the MQW structure can be clearly observed. When the lattice mismatch of the GaInP well layer increases to 0.5%, the intensity of the MQW satellite peaks decreases and their FWHM broadens. Upon further increasing the lattice mismatch to 1%, the satellite peak intensity is significantly reduced and the FWHM increases markedly. Compared with the base condition and experiment 1, the MQW satellite peaks in experiment 2 exhibit a pronounced leftward shift in the direction of compressive stress, indicating that the QW layers experience greater compressive stress. According to Equation (1), the stress in the MQW is determined by the stresses of the QW and QB layers and their respective thicknesses. Calculations show that the base condition and experiment 1 have stresses of 0% and 0.2%, respectively, while the overall stress in experiment 2 reaches 0.36%, significantly higher than that in the base condition and experiment 1. Consequently, the MQW peak position undergoes a notable leftward shift [8].

(1) $$MQW\left(Strain\right)=\frac{QW\left(Strain\right)\times QW\left(THk\right)+QB\left(Strain\right)\times QB(THk)}{QW\left(THk\right)+QB(THk)}$$

Compared with the Base condition and experiment 1, experiment 2 exhibits a noticeable reduction in the intensity of the MQW satellite peaks and a marked broadening of FWHM. According to the principles of X-ray diffraction, changes in crystal growth flatness, particularly increased interface undulation, cause deviations in the Bragg diffraction angle, resulting in a range of diffraction angles rather than a single angle. Consequently, the diffraction peaks display larger FWHM values. For the GaInP quantum well layers, an increase in In composition leads to compositional inhomogeneity, characterized by In-rich and In-poor regions. The fluctuations in In composition degrade the growth flatness of the MQWs, an effect that becomes more pronounced at higher In concentrations. This phenomenon also explains the observed increase in the spectral linewidth.

### 3.2. Influence of Well-Layer Strain on the Optoelectronic Performance of Micro-LEDs with Different Pixel Sizes

**Figure 4:**
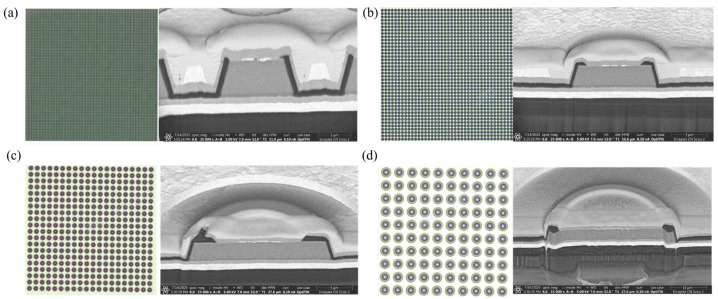
Micro-LED arrays and FIB cross-sectional images: (**a**) 10 μm pixel pitches; (**b**) 20 μm pixel pitches; (**c**) 50 μm pixel pitches; (**d**) 100 μm pixel pitches.

Figure 4 shows optical images of Micro-LED arrays fabricated from epitaxial wafers with different GaInP well-layer In compositions, together with focused ion beam (FIB) cross-sectional images of individual pixels. The pixel pitch diameters are about 10, 20, 50, and 100 μm. And the actual pixel sizes are about 5, 10, 25, 50 μm. Specifically, Figure 4a corresponds to 10 μm pixel pitches arranged in an 80 × 80 array; Figure 4b shows 20 μm pixel pitches in a 40 × 40 array; Figure 4c shows 50 μm pixel pitches in a 20 × 20 array; and Figure 4d shows 100 μm pixel pitches in a 10 × 10 array.

**Figure 5:**
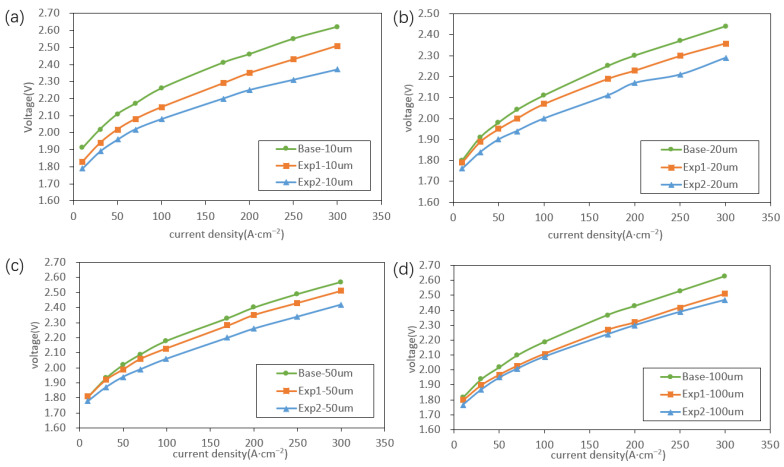
Forward current–voltage characteristics for Micro-LEDs with different pixel pitches: (**a**) 10 μm pixel pitches; (**b**) 20 μm pixel pitches; (**c**) 50 μm pixel pitches; (**d**) 100 μm pixel pitches.

Current–voltage (I–V) and external quantum efficiency–current (EQE–I) characteristics of the Micro-LED arrays were measured by electroluminescence (EL) for all three epitaxial conditions. For each pixel size, measurements were conducted over the same current density range of 10–300 A cm^−2^. The resulting I–V curves are shown in Figure 5. For all pixel pitches, the increase in In composition in GaInP leads to a gradual reduction in the forward voltage, with this effect being most pronounced for smaller devices. For 10 μm pitches, when the well-layer strain reaches 1%, the forward voltage decreases by approximately 0.1–0.3 V compared with the base condition over the measured current density range. This reduction can be primarily attributed to the decrease in bandgap energy (E_g) associated with increased In incorporation, which lowers the theoretical minimum voltage (E_g/e) of the LED. In addition, to maintain the same peak emission wavelength, the well thickness is reduced with increasing In composition, which decreases the internal resistance of the MQW region and further contributes to the lower forward voltage.

**Figure 6:**
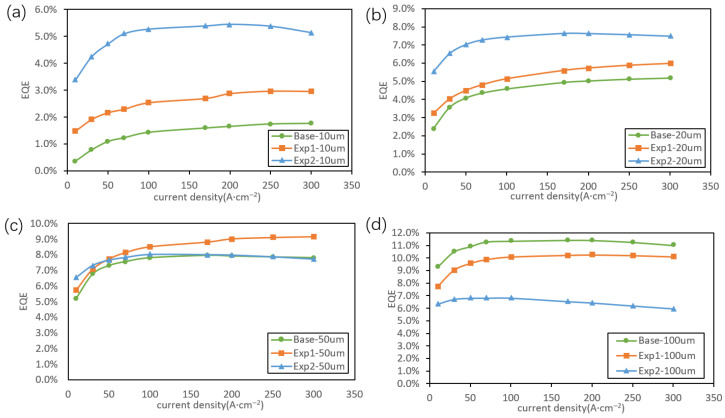
EQE–current characteristics for Micro-LEDs with different pixel sizes: (**a**) 10 μm pixel pitches; (**b**) 20 μm pixel pitches; (**c**) 50 μm pixel pitches; (**d**) 100 μm pixel pitches.

Figure 6 shows the EQE–current characteristics for Micro-LEDs of different pixel sizes. For 10 μm pitches, the EQE improves significantly with increasing In composition in the GaInP well-layer. At a strain level of 1%, the EQE is more than three times higher than that of the base condition at the same current density. For 20 μm pitches, enhanced EQE performance is still observed with increasing well-layer strain; at 1% strain, the EQE is more than 1.5 times higher than that of the base condition. When the pixel pitch increases to 50 μm, a 1% well-layer strain yields an EQE comparable to that of the base condition, while the optimal EQE is achieved at a strain level of 0.5%. Notably, for 100 μm pitches, increasing the well-layer strain results in a lower EQE, and the lattice-matched base condition exhibits the highest EQE. These results demonstrate that the well-layer strain in the MQW active region has a pronounced impact on device performance and must be optimized according to pixel size. For pixel sizes below 50 μm, increased well-layer strain is favorable, whereas for larger devices, reduced well-layer strain is preferred. This phenomenon can be explained by the following mechanism: as shown in the preceding results, increasing the In composition in the well leads to compositional fluctuations within the material, resulting in In-rich and In-poor regions. This microscopic inhomogeneity localizes carriers in localized states, restricting their lateral transport in the two-dimensional plane. Consequently, carriers are less likely to reach the sidewall defects, thereby reducing non-radiative recombination at the sidewalls and effectively increasing radiative recombination. In contrast, for larger devices, sidewall effects become negligible, and the external quantum efficiency is primarily governed by efficient current spreading and the relatively thicker quantum wells, which dominate carrier confinement and recombination.

### 3.3. Effect of Well-Layer Strain on Device Aging Characteristics for Different Pixel Sizes

Micro-LED arrays fabricated from epitaxial wafers with different well-layer strain conditions were packaged using TO headers and subjected to accelerated aging tests under a current density of 70 A cm^−2^ at 60 °C and 90% relative humidity for 500 h. During aging, the optical output power of the Micro-LED arrays was periodically measured using an integrating sphere-based optoelectronic parameter analyzer. Output power data were recorded at aging times of 48, 96, 168, 360, and 500 h.

**Figure 7:**
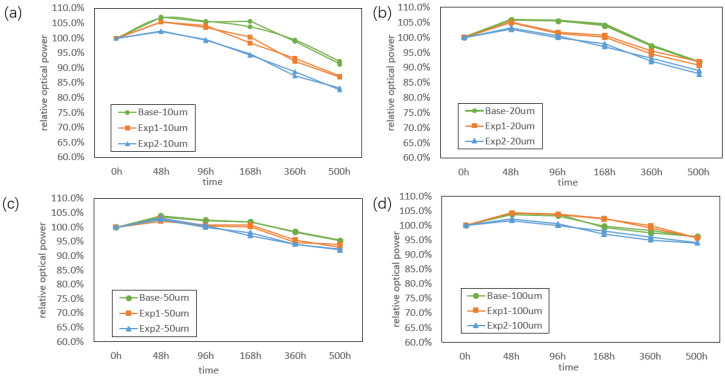
Optical power degradation curves during aging tests:(**a**) 10 μm pixel pitches; (**b**) 20 μm pixel pitches; (**c**) 50 μm pixel pitches; (**d**) 100 μm pixel pitches.

Figure 7 presents the normalized relative optical output power as a function of aging time for Micro-LEDs with different pixel sizes and well-layer strain conditions (two samples were tested for each condition). For 10 μm pitches, the optical power degradation increases with increasing GaInP well-layer strain. At a strain level of 1%, the optical output power decreases to approximately 85% after 500 h, representing an additional 5% degradation compared with the base condition, which retains about 90% of its initial output power. When the pixel pitch increases to 20 μm, the difference in optical power degradation among different strain conditions becomes smaller, although the base condition still exhibits the lowest degradation. For pixel pitches of 50 and 100 μm, the optical power degradation is nearly independent of the well-layer strain, with all samples showing less than 5% degradation after 500 h.

These results indicate that optical power degradation becomes more pronounced in devices with smaller pixel dimensions and higher In composition in the quantum wells. This is likely associated with the enhanced non-radiative recombination at the sidewalls in smaller devices. Additionally, the increase in indium content and the appropriate reduction in well thickness result in an increased energy separation between the quantized states (ground and excited states) within the well. At elevated temperatures, the confinement capability of the well for carriers occupying these higher energy states deteriorates, leading to carrier leakage and, consequently, a degradation in reliability performance.

## 4. Conclusions

In this work, AlGaInP/GaInP multiple quantum well (MQW) Micro-LED epitaxial wafers with different In compositions in the quantum-well layers were grown by metal–organic chemical vapor deposition (MOCVD) and subsequently fabricated into Micro-LED display arrays with pixel pitches of 10 µm, 20 µm, 50 µm, and 100 µm. The results demonstrate that introducing a compressive strain of 1% into the GaInP wells significantly enhances the EQE of 10 µm pitch Micro-LEDs. Compared with lattice-matched well growth, the forward voltage is reduced by 0.1–0.3 V and the EQE increases by approximately threefold, while maintaining more than 85% of the initial optical output after 500 h of aging. However, as the device size increases, lattice-matched well growth yields higher EQE, indicating that MQW strain requires size-dependent optimization.

These findings provide important insights into the design and epitaxial growth of both Micro-LEDs and large-area LEDs, highlighting the critical role of well-layer strain engineering in achieving optimal device performance across different device dimensions.

## Figures and Tables

**Table 1:** Growth parameters of GaInP well layers.

Experimental Conditions	Well in Composition	Well Thickness (nm)	Well Lattice Mismatch (Δa/a)	Barrier in Composition	Barrier Thickness (nm)
Base	0.5	6	0%	0.5	7
Experiment 1	0.55	5	0.5%	0.5	7
Experiment 2	0.6	4	1%	0.5	7

## Data Availability

The original contributions presented in the study are included in the article. Further inquiries can be directed to the corresponding author.

## Abbreviations

The following abbreviations are used in this manuscript:

Cp_2_Mg	Bis(cyclopentadienyl)magnesium
EL	Electroluminescence
EQE	External quantum efficiency
FIB	Focused ion beam
FWHM	Full width at half maximum
HRXRD	High-resolution X-ray diffraction
ICP	Inductively coupled plasma
IQE	Internal quantum efficiency
ITO	Indium tin oxide
I–V	Current–voltage
LED	Light-emitting diode
MOCVD	Metal–organic chemical vapor deposition
MQW	Multiple quantum well
PL	Photoluminescence
SC-MQW	Strain-compensated multiple quantum well
TMAl	Trimethylaluminum
TMGa	Trimethylgallium
TMIn	Trimethylindium
XRD	X-ray diffraction
